# Dynamic cluster structure and predictive modelling of music creation style distributions

**DOI:** 10.1098/rsos.220516

**Published:** 2022-11-02

**Authors:** Rajsuryan Singh, Eita Nakamura

**Affiliations:** ^1^ Music Technology Group, Universitat Pompeu Fabra, Barcelona 08002, Spain; ^2^ The Hakubi Center for Advanced Research, Kyoto University, Kyoto 606-8501, Japan; ^3^ Graduate School of Informatics, Kyoto University, Kyoto 606-8501, Japan

**Keywords:** cultural evolution, statistical learning, music creation style, dynamical system, social dynamics, trend prediction

## Abstract

We investigate the dynamics of music creation style distributions to understand cultural evolution involving intelligence to create complex artefacts. Previous work suggested that a music creation style can be quantified as statistics describing a generative process of music data, and that the distribution of music creation styles in a society has cluster structure related to the presence of different musical genres. To find patterns in the dynamics of the cluster structure, we analysed statistics of melodies in Japanese popular music data and statistics of audio features in American popular music data. Using statistical modelling methods, we found that intra-cluster dynamics, such as the contraction and the shift of a cluster, as well as inter-cluster dynamics represented by clusters’ relative frequencies, often exhibit notable dynamical modes. Additionally, to compare the individual contributions of these different dynamical aspects for predicting future creation style distributions, we constructed a fitness-based evolutionary model and found that the predictions of cluster frequencies and cluster variances often have comparable contributions. Our results highlight the relevance of intra-cluster dynamics in music style evolution, which have often been overlooked in previous studies. The present methodology can be applied to cultural artefacts whose generative process can be characterized by a discrete probability distribution.

## 1. Introduction

The theory of cultural evolution uses mathematical models to understand how knowledge and intelligent behaviours are shared and developed in human society [1,2]. These models represent an evolutionary process where a cultural trait (knowledge, behaviour, etc.) is transmitted over generations, with possible modifications and selection biases [3–6], similarly as in models of biological evolution [7,8]. Some creative cultures, such as science and the arts, are developed through the ability to learn to create complex artefacts. Therefore, a quantitative understanding of the dynamic changes in the knowledge for creating artefacts is essential to uncover the mechanisms of the evolution of human’s highly developed cultures. Owing to its significance and universal presence in society [9], music has been a popular topic in cultural evolution research [10–19]. Here, we study the evolution of music styles using large-scale data analysis and computational modelling.

Mathematical representation of knowledge for music composition has long been studied in the field of information science and some other fields. Currently, a successful model of the knowledge acquision process is the statistical learning model, where the knowledge is represented as statistics extracted from musical products [20,21]. For example, a number of automatic music composition systems capable of generating songs similar to human-made ones have been realized based on statistical learning [22,23]. Additionally, cognitive scientific experiments have also indicated that humans employ statistical learning to acquire knowledge of music and language [24,25]. Moreover, statistical learning has been used as a model to explain how humans learn to understand music in tasks such as music expectation [26], genre classification [27] and musical analysis [28]. Finally, systematic knowledge of music composition taught in music classes can be partly incorporated in musical statistics; for example, prohibition of a certain melodic motion in harmony theory can be described as a zero or very small probability assigned to that motion. It has also been shown that systematic knowledge presented in music textbooks cannot be computationally formalized in a straightforward manner and is insufficient to realize a flexible and high-quality automatic music generation system (e.g. [29]).

From the perspective of statistical learning, music creation is described as a process of generating time series data using a probabilistic model, with the knowledge used in this process represented by the statistical parameters of the model. For example, in the Markov model, a standard model for melody generation [30], the parameters represent the transition probabilities of pitches or other musical elements. This formalization enables quantitative comparisons of creation styles between songs or composers, and accordingly allows a mathematical description of the macroscopic (social-level) dynamics of the creative culture in terms of the time evolution of the distribution of the statistical parameters. In the following, we identify these statistical parameters as quantified creation styles.

Musicologists have studied how musical features have changed over time. Traditionally, researchers have provided detailed accounts of composition techniques within the social context [31–34]; however, most analyses lacked quantitative formulations that enable prediction and hypothesis testing. Owing to the increasing availability of digital music data and computational analysis methods, large-scale quantitative analyses of music evolution have recently become possible. Previous studies have confirmed that in the history of Western classical and popular music, the means of various musical features evolved steadily and directionally [10–12,14–16]. Further, these evolutions were punctuated by periods of rapid change (i.e. revolutions) [11,12,14], justifying the widely accepted view of music history as a succession of distinct eras (e.g. Romantic Period [32], Rock Era [33], etc.).

Nonetheless, from the viewpoint of modelling the music creation process, however, it is difficult to represent the distributions of musical features of each time period simply by their means [12,14]. This is because there are typically multiple clusters (genres, modes, etc.) of musical styles coexisting concurrently in a society, and averaging the features of different clusters is not a logical approach in most practical cases. For example, classical composers used both the major and minor scales, which can be represented as different probability distributions over pitches, but did not use the hybrid structure obtained by averaging these probability distributions. To address this internal structure of musical feature distributions, previous studies [12,14] have applied data-driven clustering methods and analysed the evolution of the frequencies of the obtained clusters. The clusters often corresponded to known genres or interpretable composition styles, and the revolutions in musical styles were often associated with the rise and decline of particular clusters (inter-cluster dynamics). These results suggest that the distribution of music creation styles consists of concurrent and transient clusters, where the evolution of the distribution is caused by the dynamic changes of the clusters’ relative frequencies.

The concurrent and transient cluster structure in music evolution raises further questions. First, what internal (intra-cluster) dynamics do clusters have? As jazz music, for example, is categorized into subgenres associated with certain time periods [35], musical genres are often considered to have a hierarchical and dynamic structure. In musicology, changes in the creation style of individual composers (e.g. Beethoven [32]) are also studied. This question is important for evolutionary modelling because intra-cluster dynamics imply that a content-based artefact-level selective pressure is at work, in addition to the group-level selection implied by inter-cluster dynamics. Second, to what extent can we predict the future distribution of music creation styles by incorporating the cluster dynamics into an evolutionary model? To quantitatively study the relative contributions of the inter- and intra-cluster dynamics, it is necessary to develop a computational model that can infer and predict the inter- and intra-cluster dynamics from data.

To address these questions, we conduct evolutionary analyses of music data using statistical modelling methods. We use two datasets of Japanese and American popular music, both containing songs appearing in top sales charts covering 50 years or more (the United States (US) and Japan had the largest music recording industries globally during the 2010s, according to the reports by the International Federation of the Phonographic Industry [36]). We extract musical statistics from these data, apply clustering and study the dynamic changes in the clusters’ structure. We observe some intra-cluster dynamics, such as changes in the clusters’ means and variances. We show that these dynamics can be described by a statistical model called the dynamic Dirichlet mixture model (DDMM), whereby the intra- and inter-cluster dynamics are translated into the time-evolving parameters of the DDMM in a unified manner. We then formulate an evolutionary model for the DDMM parameters in which the fitness-based competitive dynamics of the clusters and those of musical elements within each cluster are incorporated. Finally, we develop a method for inferring and predicting these parameters and quantitatively examine the contributions of the inter- and intra-cluster dynamics to predict the future distribution of music creation styles.

The remainder of the paper is structured as follows. In §2, we analyse Japanese popular music data and the dynamics of cluster structure. In §3, we develop our predictive model. Section 4 presents the results for the analysis of US popular music data. We summarize the findings and discuss their implications in §5.

## 2. Dynamics of creation style distributions

In this section, we analyse the cluster structure in Japanese popular music data, finding that some clusters exhibit notable intra-cluster dynamics. We also point out that major aspects of cluster dynamics can be described by the DDMM.

### 2.1. Data and analysed musical statistics

We used a dataset of Japanese popular songs (J-pop dataset) that comprised 1399 songs in the top sales charts provided by Oricon Inc.; the charts were mainly based on the number of phonograph record and CD sales. The vocal/main melodies of all songs that ranked within the top 20 in the yearly charts between 1950 and 2019 were transcribed and encoded in the MusicXML format (except for one song whose audio file was not accessible). Only notes in the main melody were analysed for instrumental music and songs with duets or a chorus, and notes with unclear pitches as in rap music were not included. For computational analysis, each melody was represented as a sequence of pairs of integers, one representing the pitches and the other representing the onset times of musical notes (rests were not used for the analysis). The pitch of a note was represented in units of semitones, and all songs were transposed to the C major or A minor key prior to the analysis. The onset time of a note was represented as an integer *b* ∈ {0, 1, …, 47} corresponding to its relative position in a bar (called the metrical position). For example, *b* = 0 indicates the downbeat position, and in 4/4 time, *b* = 12 indicates the second beat position. We fixed the temporal resolution such that a bar has 48 units, and notes with onset times that cannot be expressed in the resolution were excluded from the analysis.

For musical statistics, we used the pitch and rhythm bigram probabilities to represent the creation style of each song. These bigram probabilities are equivalent to the transition probabilities of Markov models and can describe different musical scales and rhythmic modes [30]. To obtain pitch bigrams, we used the extended pitch class representation, in which a transition from pitch *p*′ to pitch *p* is represented as a bigram (*q*′, *q*) (0 ≤ *q*′ ≤ 11 and 0 ≤ *q* ≤ 35) given by *q*′ ≡ *p*′ (mod 12) and *q* ≡ *q*′ + *p* − *p*′ (mod 36). Thereby, we obtained a representation independent of the pitch range and capable of discriminating pitch intervals between −17 and 17 semitones (an interval of 18 semitones and that of −18 semitones are identified). Thus, the pitch bigram probabilities had 12 × 36 elements. The rhythm bigram probabilities were calculated from the frequencies of the bigrams of metrical positions; they had 48 × 48 elements. Although we need both of the probabilities for pitches and metrical positions to construct a statistical model for melody generation, we treated them separately in the following analysis to make interpretation of the results easier.

After representing each song *n* with a tuple $\left(\theta_{n},t_{n}\right)$ of the probability vector $\theta_{n}$ of its pitch/rhythm statistics and its created time *t*_*n*_, we applied a clustering method based on the discrete distribution mixture model. Specifically, we used the expectation–maximization (EM) algorithm [37] with random initialization to train *K* sets of discrete distributions representing the clusters. After convergence, each song was assigned to the cluster with the maximal likelihood. Created time information was not used in the clustering process.

### 2.2. Qualitative analysis of dynamics of creation style distributions

To obtain an intuitive overview of the creation style distribution dynamics, we represented the probability vectors of songs in a two-dimensional space using the *t*-distributed stochastic neighbour embedding (*t*-SNE) visualization method [38]. For this visualization, we first calculated the Jensen–Shannon (JS) divergence between each pair of probability vectors, which measures their ‘distance’. Then, the *t*-SNE method was used to map the probability vectors into the visualization space where the distances were retained as much as possible.

**Figure 1.**
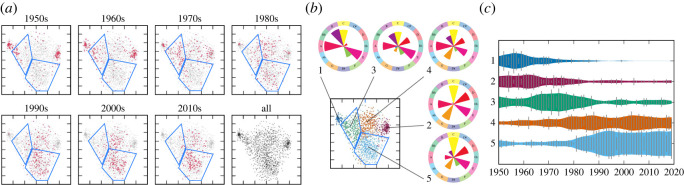
The evolution of the distribution and cluster structure of the pitch statistics in the J-pop data. (*a*) The two-dimensional visualization of the data distribution (blue polygons outline regions corresponding to clusters 3 and 5). (*b*) Result of the cluster analysis. The figures in circles represent the pitch-class distributions, where 12 divided sectors correspond to pitch classes in a clockwise order (colours are arbitrary) and probability values are indicated by the sizes of pie-shaped objects. (*c*) The evolution of the relative frequencies of the clusters.

Before discussing the dynamics, we first discuss the cluster structure of the creation style distributions using the obtained visualization. From the results for the pitch statistics in figure 1*a*, we can find a small cluster on the left and another small cluster on the right, as well as a large cluster spread in the central region. Inside the large cluster, we can find some structures of density variations, but there are no clearly separated clusters. The clustering method, assuming five classes (*K* = 5), automatically identified the two small clusters and divided the large cluster into three smaller clusters (figure 1*b*). The average statistics for the clusters, represented as pitch-class probabilities, show that all five clusters can be associated with distinguishable musical scales. Cluster 1 can be associated with a minor pentatonic scale (known as Japanese *in* scale [39]), cluster 2 with a major pentatonic scale, cluster 3 with a minor hexatonic scale, cluster 4 with the minor diatonic scale, and cluster 5 with the major diatonic scale. These musical scales, except the hexatonic scale, match musical scales documented by musicologists studying Japanese popular music [40]. From a macroscopic viewpoint, clusters 3, 4 and 5 form a family of diatonic scales. These clusters in addition to the larger cluster that includes them form a hierarchical structure in the creation style distribution.

**Figure 2.**
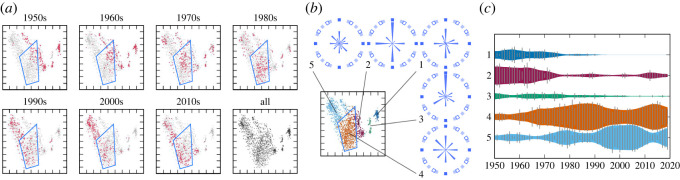
The evolution of the distribution and cluster structure of the rhythm statistics in the J-pop data. (*a*) The two-dimensional visualization of the data distribution (blue polygons outline regions corresponding to cluster 4). (*b*) Result of the cluster analysis. The figures in circles represent the distributions of metrical positions, where the circle period corresponds to a bar, metrical positions are ordered clockwise, and probability values are indicated by the sizes of wedge-shaped objects. (*c*) The evolution of the relative frequencies of the clusters.

Similar observations can be drawn from the results for the rhythm statistics. From figure 2*a*, we find three clearly separated small clusters on the right and a large cluster with visible internal structures on the left. Assuming five classes, the clustering method identified three clusters inside the large cluster and assigned two clusters to the separated clusters on the right (figure 2*b*). Again, we can musically interpret the five clusters using their average statistics, represented as metrical position probabilities. Cluster 1 can be associated with a dotted rhythm, cluster 2 with a four-beat rhythm, cluster 3 with ternary metres, cluster 4 with an eighth-note rhythm and cluster 5 with a 16th-note rhythm. This creation style distribution also has a hierarchical structure with a large cluster containing smaller clusters that overlap with each other.

These results provide some implications for the applicability of representing creation style distributions as a mixture of clusters. On the one hand, representation using clusters can capture the internal structure of a creation style distribution more precisely than representing it as a simple (e.g. unimodal) distribution, and to some extent, it can reproduce classifications of musical patterns studied by musicologists. On the other hand, the clusters often overlap significantly and possibly form a hierarchical structure, which invalidates the notion of definitive clusters. Despite these limitations, cluster representation is still effective for approximating the complex structure of creation style distributions using simple and tractable mathematical models and interpreting the results.

We now discuss the dynamics of creation style distributions. From figures 1*a* and 2*a*, we find drastic changes in the creation style distributions for both statistics. For the pitch statistics, most songs were located in the leftmost or rightmost regions in the 1950s and 1960s, and in the central region in the 1990s and later. This overall change is clearly visible in the evolution of cluster frequencies, which shows the dominance of clusters 1 and 2 in the early years and the dominance of clusters 4 and 5 in later years (figure 1*c*). Similarly, for rhythm statistics, most songs were located in the upper-right region in the panels for the 1950s and 1960s, and in the lower-left region in the panels for the 1980s and later. The evolution of cluster frequencies in figure 2*c* also reflects this overall transition. Therefore, the major transitions in the creation style distributions of Japanese popular songs can be captured by the inter-cluster dynamics, similar to the observations made in the evolution of US popular music [12] and Western classical music [14] (see also §4).

We also observed some dynamics within the clusters. First, the distribution of the pitch statistics in cluster 5 reduced its variance from the 1990s to the 2010s. This contraction of the cluster is ascribed to the movement towards market concentration during the period: 103 distinct artists appeared on the chart in the 1990s, whereas only 76 (resp. 32) distinct artists appeared in the 2000s (resp. the 2010s). Second, the distribution of pitch statistics in cluster 3 exhibits a significant shift of a concentrated region from the 1950s to the 1980s. This reflects the movement of musical preferences from the minor pentatonic scale to the minor diatonic scale in songs that appear on the chart. A similar shift of a concentrated region can be observed in cluster 4 of the rhythm statistics between the 1960s and the 1980s. This shift represents the movement towards widely using syncopated rhythms (see electronic supplementary material).

These observations show that the creation style distributions exhibit dynamical modes that cannot be purely represented as changes in the frequencies of clusters having stationary distributions. To capture the collective nature of a contraction or shift of a cluster, we should consider the intra-cluster dynamics, or equivalently, represent the cluster by its smaller components and consider their coherent frequency dynamics that also depend on their content features. Therefore, the present analysis indicates that both inter- and intra-cluster dynamics are relevant for an effective modelling of the evolution of music creation style distributions.

### 2.3. Quantitative analysis of intra-cluster dynamics using Dirichlet distribution

While the qualitative analysis in the previous section was intuitive, the two-dimensional visualization should be seen as an approximate picture of the distribution of creation styles, which were in fact represented as high-dimensional probability vectors. To quantitatively analyse intra-cluster dynamics in the creation style distribution, we need to mathematically formulate clusters and describe their attributes, such as the size and centre position, in the space of probability vectors. Note that a standard statistical formulation uses the Gaussian distribution to represent a cluster in the Euclidean space [37]. The Gaussian distribution has parameters that can represent the basic properties of a cluster (the mean vector represents the cluster’s centre while the covariance matrix represents the cluster’s size and direction) and is well-defined in the Euclidean space. By extending this idea to the case of our concern, we can use the Dirichlet distribution, which is commonly used in Bayesian statistics to represent a cluster in the space of probability vectors [37]. It is also known that the Dirichlet distribution can approximately reproduce the distribution of the Shannon entropies for rhythm statistics in popular music melodies [41].

For *D*-dimensional probability vectors $\theta ={\left(\theta_{i}\right)}_{i=1}^{D}$ $\left(\sum_{i}\theta_{i}=1\right)$, the Dirichlet distribution is defined as

2.1 $$\mathrm{Dir}(\theta ;\alpha ,\mu )=\Gamma (\alpha )\prod_{i=1}^{D}\frac{\theta_{i}^{\alpha \mu_{i}-1}}{\Gamma (\alpha \mu_{i})},$$

where the parameter *α* > 0 represents the concentration and the probability vector $\mu ={\left(\mu_{i}\right)}_{i=1}^{D}$ is the mean (or base) distribution. A Dirichlet distribution is a probability distribution of probability vectors, and the component-wise mean and variance are given as *E*(*θ*_*i*_) = *μ*_*i*_ and *V*(*θ*_*i*_) = *μ*_*i*_(1 − *μ*_*i*_)/(*α* + 1), respectively. Thus, the mean μ can be interpreted as the centre of the cluster, and the concentration *α* is a parameter related to the cluster’s size (the variance is inversely proportional to *α* + 1). Given a set of probability vectors assigned to a cluster, we can estimate these parameters for the cluster using the maximum likelihood estimation method [42].

**Figure 3.**
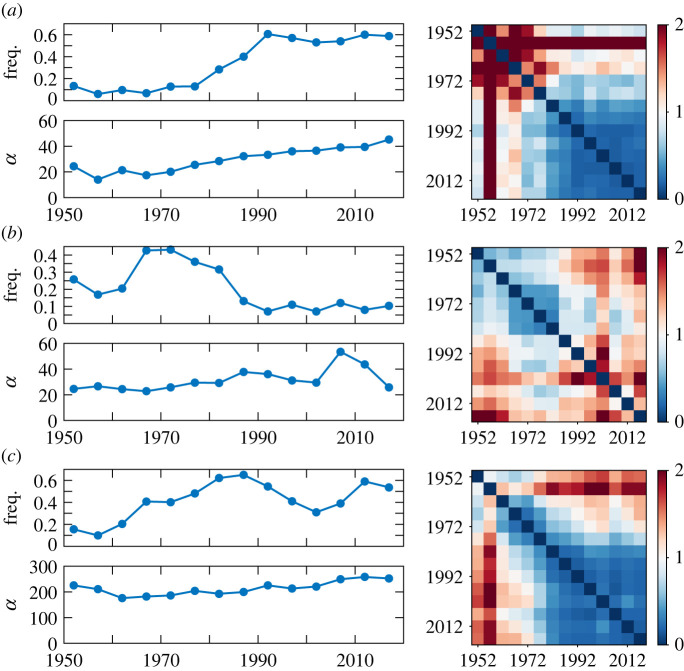
The evolutions of the relative frequency, concentration and mean distribution for (*a*) cluster 5 and (*b*) cluster 3 of the pitch statistics in figure 1 and for (*c*) cluster 4 of the rhythm statistics in figure 2. The evolution of the mean distribution is represented by the symmetric Kullback–Leibler (SKL) divergences between time slice pairs on the right.

Figure 3 shows the evolution of the estimated parameters of the Dirichlet distributions for some clusters computed after the data samples were divided into time slices. To quantify the temporal differences in the mean distributions, we used the symmetric Kullback–Leibler (SKL) divergence, which is a distance measure for general probability distributions. Figure 3*a* illustrates the evolution of cluster 5 of the pitch statistics; the contraction of the cluster from the 1990s to the 2010s is represented as an increase in the concentration parameter. Note that the SKL divergences of the mean distributions between any pair of time slices remained far below unity during this time period, indicating that the shift of the cluster centre was relatively small. Figure 3*b* illustrates the evolution of cluster 3 of the pitch statistics; the shift of the cluster centre from the 1950s to the 1980s is reflected in the relatively large off-diagonal elements in the SKL divergence matrix during this period. Similarly, in figure 3*c*, which illustrates the evolution of cluster 4 of the rhythm statistics, the off-diagonal elements in the SKL divergence matrix for the period between the 1960s and the 1980s take considerably larger values than those for the period between the 1990s and the 2010s, quantitatively demonstrating that a shift of the cluster centre occurred in the early years.

These results show that the Dirichlet distribution can be used to quantitatively analyse the intra-cluster dynamics of the creation styles. In particular, a contraction (and possibly expansion) and shift of the centre of a cluster can be described as evolutions of the concentration and mean distribution, respectively. Moreover, as shown in the next section, together with a dynamical model for the parameter evolution, the Dirichlet distribution can be used as a statistical model for predicting the future creation style distribution. This formulation also allows us to evaluate the predictive accuracy by measuring the likelihood, following the general statistical framework.

## 3. Predictive modelling

### 3.1. Dynamic Dirichlet mixture model

In §2, we found that both inter- and intra-cluster dynamics are important aspects of the evolution of music creation style distributions. Here, we construct a model incorporating these dynamics to predict future creation style distributions and examine the relevance of intra-cluster dynamics for predictive modelling. As explained in §2.3, the clusters of creation styles in the space of probability vectors can be described by Dirichlet distributions. Thus, the distribution of creation styles composed of multiple clusters can be described by a Dirichlet mixture model (DMM) that is defined as

3.1 $$P(\theta ,t)=\sum_{k=1}^{K}\pi_{k}(t)\;\mathrm{Dir}(\theta ;\alpha_{k}(t),\mu_{k}(t)),$$

where θ represents a probability vector as in equation (2.1), *k* indexes clusters and *K* denotes the number of clusters. The quantity *π*_*k*_ (called the mixture probability) represents the relative frequency of cluster *k* (we have $\sum_{k}\pi_{k}=1$).

In equation (3.1), the dynamics of the creation style distribution are decomposed into the time evolutions of the parameters on the right-hand side, which is the basic assumption of the present model. The inter-cluster dynamics are incorporated in the time-dependent mixture probabilities *π*_*k*_(*t*), representing the changes in the cluster frequencies. The intra-cluster dynamics are incorporated in the time-dependent means $\mu_{k}(t)$, representing the shifts of the cluster centres, and the time-dependent concentration parameters *α*_*k*_(*t*), representing the contractions and expansions of the clusters. Hereafter, the model in equation (3.1) is called the DDMM.

To use the DDMM for predicting future creation style distributions, given data up to the present, two problems should be addressed. The first is the parameter estimation problem, that is, estimating the parameters *π*_*k*_(*t*), *α*_*k*_(*t*) and $\mu_{k}(t)$ at each time point up to the present that optimally fit the given data. The second is the parameter prediction problem, that is, predicting future values of the parameters given the parameter values up to the present. We discuss the first problem in §3.2 and the second problem in §3.3. As a problem set-up, we consider the situation in which the data samples (musical statistics) are assigned to one of the clusters, as in §2.

### 3.2. Parameter estimation problem

A simple solution to the parameter estimation problem is to learn the DDMM parameters for each year (or other units of time slice), *t*, using the data samples created in that year. The mixture probabilities *π*_*k*_(*t*) and mean distributions $\mu_{k}(t)$ can be obtained by taking the sample averages, and the concentration *α*_*k*_(*t*) can also be determined using the maximum likelihood estimation method [42]. However, this simple method suffers from the data sparseness problem when the available data are not sufficient and the number of analysed statistics is large, as is the case for the present analysis.

To address this problem, one approach is to use all data samples created by time *t* with certain weighting factors to learn the parameters. Using the standard exponential decay factor, this weighted average method can be formulated as follows. The mixture probabilities and mean distributions can be obtained as

3.2 $$\pi_{k}^{\mathrm{WA}}(t)=C{\left(t\right)}^{-1}\sum_{s=-\infty }^{t}\;e^{(s-t)/\tau }\pi_{k}^{S}(s)$$

and

3.3 $$\mu_{k}^{\mathrm{WA}}(t)=C{\left(t\right)}^{-1}\sum_{s=-\infty }^{t}\;e^{(s-t)/\tau }\mu_{k}^{S}(s),$$

where $\pi_{k}^{S}(s)$ and $\mu_{k}^{S}(s)$ are the values obtained using the aforementioned simple method, e^(*s*−*t*)/*τ*^ is the decay factor, *τ* is the time constant and $C(t)=\sum_{s=-\infty }^{t}\;e^{(s-t)/\tau }$ is a normalization factor. For the concentration parameter, we can use the log-likelihood obtained from a single-time log-likelihood $L_{k}^{S}(\alpha ;s)$ calculated from equation (2.1) for cluster *k*, as

3.4 $$L_{k}^{\mathrm{WA}}(\alpha ;t)=C{\left(t\right)}^{-1}\sum_{s=-\infty }^{t}\;e^{(s-t)/\tau }\;L_{k}^{S}(\alpha ;s),$$

and then estimate its value as $\alpha_{k}^{\mathrm{WA}}(t)={\mathrm{argmax}}_{\alpha }L_{k}^{\mathrm{WA}}(\alpha ;t)$.

**Figure 4.**
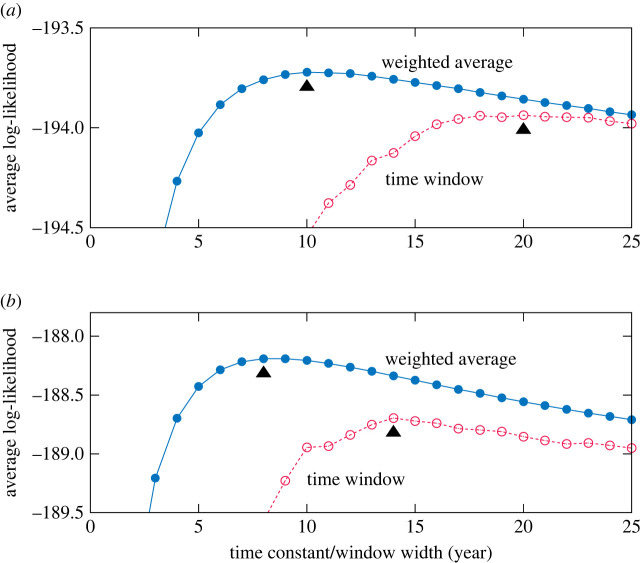
Log-likelihood of the DMMs (*K* = 5) estimated by the weighted average method and time window method, for the (*a*) pitch statistics and (*b*) rhythm statistics of the J-pop dataset.

A suitable value for the time constant *τ* can be determined from the data by optimizing the likelihood of the estimated DMM. Figure 4 shows how the likelihood depends on the value of *τ*. To obtain this result, we set the referential year (tentative ‘present year’ for the analysis) *t*_0_ to each value in the range [1960, 2018]. Then, we estimated the DMM parameters and calculated the likelihood of the data created in year *t*_0_ + 1 (assuming that the creation style distribution would not change significantly in 1 year). The likelihood was optimal at *τ* = 10 for the pitch statistics and *τ* = 8 for the rhythm statistics. We used these values in the following analysis. For comparison, the result of estimating the DMM parameters using data samples in a time window of various widths (using a 1-year width is the same as the simple method) is also shown in figure 4, demonstrating the superiority of the weighted average method.

### 3.3. Parameter prediction problem

To solve the parameter prediction problem, we construct a model for the inter- and intra-cluster dynamics within the framework of cultural evolution theory [1]. The model we construct here is a simplified description of the generative process for songs produced by creators who learn their creation styles from past creators. As shown later in this section, the outcome of this model is a statistical tool used for data analysis and prediction, rather than a tool for theoretically understanding the evolutionary process. However, we explain how the model attempts to capture the elements of the underlying process, to clarify the simplifications and abstractions made during the model construction and to make this paper more useful for those who wish to develop more refined models or apply the method to other cultural domains or in other situations.

Each creator in our model has a set of cultural traits (k,θ), where *k* represents the cluster to which it belongs and θ represents the musical statistics describing its creation style. With its statistics θ a creator produces songs, which are presented to the imaginary society of the model and cause the creator’s evaluation. Although a creator can produce multiple songs in reality, in our model a creator produces only one song and the song’s statistics are identified with the creators statistics, since we do not analyse artist labels for the songs of the datasets. Therefore, creators and songs are effectively the same thing in our model, but we consider creators (not songs) as replicating organisms in the sense of evolution theory.

At each year (the generation time of our model) a population of new creators is introduced, and each new creator inherits its cultural traits from a creator in the previous generation. The inheritance process is described in two steps. First, a new creator *n* selects a cluster *k*_*n*_ with a probability proportional to the cluster’s relative frequency weighted by the fitness $w_{k_{n}}$ of the cluster. This fitness represents the inclusive effect of selective pressure resulting from creators’ preferences, the public’s evaluation, and other possible selection biases (such as the effect of advertisement and reputation [43]) on the cluster. The fitness drives the inter-cluster dynamics. Next, the new creator selects a cultural parent among the creators of the previous generation belonging to cluster *k*_*n*_ and inherits the parent’s statistical traits. The cultural parent $\tilde{n}$ is selected with a probability proportional to the fitness $w(\theta_{\tilde{n}},k_{n})$ of its statistical traits $\theta_{\tilde{n}}$. This fitness represents the inclusive effect of creators’ preferences etc. on the musical elements and is defined for each selected cluster *k*_*n*_. The fitness drives the intra-cluster dynamics.

As in the models in evolutionary biology [7,8], iterations of these processes gradually change the distribution P(k,θ,t) of the clusters and the statistical traits within the population of creators at time *t*. For mathematical simplicity, we consider an infinite-size population model, in which case the distribution is a continuous function of θ. We also assume that the distribution has the form $P(k,\theta ,t)=\pi_{k}(t)\;\mathrm{Dir}(\theta ;\alpha_{k}(t),\mu_{k}(t))$, as given in equation (3.1), which can be realized by a suitable choice of initial condition and form of the fitness function w(θ,k).

To use this evolutionary model for the prediction problem, it is necessary to estimate fitness from past data. Since such evolutionary processes are usually stochastic, while we can only observe a finite (and often small) number of data samples, statistical noise should be considered for the fitness estimation. In addition, as musical preferences themselves are also likely to change over time [15], a model that allows extensions to incorporate the evolution of fitness is needed. In the following paragraphs, we formulate a fitness-based model within a statistical framework that allows a robust estimation of fitness and extensions to dynamically changing fitness.

We first present a model for predicting the evolution of mixture probabilities *π*_*k*_(*t*). The basic equation of fitness-based evolutionary dynamics [7] can be represented as

3.5 $$\pi_{k}(t+1)=\frac{w_{k}\pi_{k}(t)}{\bar{w}},$$

or

3.6 $$\ln\;\pi_{k}(t+1)=\ln\;\pi_{k}(t)+\ln\left(\frac{w_{k}}{\bar{w}}\right),$$

where *w*_*k*_ denotes the fitness (reproduction rate) for cluster *k*, and the average fitness $\bar{w}$ is defined as $\bar{w}=\sum_{\ell }w_{\ell }\pi_{\ell }(t)$. The second equation, which is a simple arrangement of the first equation, can be viewed as the equation of motion for the log probabilities, where the logarithm of the relative fitness $w_{k}/\bar{w}$ corresponds to the velocity.

There are two points to be discussed before moving forward. First, although the relative fitness depends on all *π*_ℓ_(*t*) through $\bar{w}$, the effect of the other *π*_ℓ_(*t*) on the dynamics of *π*_*k*_(*t*) is small if the speed of evolution is slow. In this approximation of slow evolution, which we adopt in this study, the dynamics of *π*_*k*_(*t*) are decoupled from each other and can be treated independently (until they are finally normalized after solving the dynamics). Second, the above equations describe a model for a large population and do not include the statistical noise observed in a finite set of data samples. If we write *x*(*t*) = ln *π*_*k*_(*t*) and $v(t)=\ln(w_{k}/\bar{w})$, and represent the noise added to the observed *x*(*t* + 1) by *ε*(*t*), then the modified model is given as

3.7 x(t+1)=x(t)+v(t)+ϵ(t).

Specifically, *ε*(*t*) is described by a Gaussian noise that satisfies 〈*ε*(*t*)〉 = 0 and 〈*ε*(*t*)*ε*(*s*)〉 = *σ*^2^*δ*_*ts*_. In the approximation of a constant fitness, *v*(*t*) is independent of time.

We can extend the model to include the dynamics of the fitness by treating *v*(*t*) as a time-dependent variable and adding a dynamical equation. We obtain a natural model for *v*(*t*) by introducing a variable *η*(*t*) representing the temporal variation of *v*(*t*) as

3.8 v(t+1)=v(t)+η(t),

where *η*(*t*) is also described by Gaussian noise. In general, we can also introduce variables representing higher-order time derivatives and construct a general model (see electronic supplementary material). Equations (3.7) and (3.8) (with possible additional equations) are known as the state-space model in statistics [37]. An advantage of the present formulation is that we can apply the well-developed statistical theory of state-space models to estimate the fitness (or velocity *v*(*t*)) from the data and predict the future values of *x*(*t*). The details of this method are presented in electronic supplementary material.

We can derive the state-space models for predicting the evolutions of the mean distributions *μ*_*ki*_(*t*) and concentrations *α*_*k*_(*t*) in a similar fashion (see electronic supplementary material). In the case of mean distributions, we can interpret the fitness as representing the preferences of the creators and the public with respect to constituent musical elements indexed by *i*. For concentrations, we could not find such an intuitive interpretaton of the corresponding fitness. Nevertheless, if the evolution of the concentrations results from dynamics where their velocities vary smoothly over time, a state-space model can serve as an approximating model for the dynamics. Therefore, we apply such a model in the following analysis.

In a preliminary analysis, we found that using predictions that are more conservative than those directly obtained by the model often leads to higher accuracy. More precisely, after the velocity is predicted by the model, it is reduced by a factor called the fitness reduction factor before it is used for making predictions. In our analysis, the reduction factor was optimized for each set of parameters (mixture probabilities, concentrations and mean distributions) in the range [0.01, 1]. See electronic supplementary material for details.

### 3.4. Evaluation of predictive accuracies

To evaluate the predictive model for creation style distributions, we conducted two computational experiments. In the first experiment, we evaluated the predictive accuracies of the DDMM parameters by component and compared them with other possible methods to examine the effectiveness of the fitness-based evolutionary model. In the second experiment, we evaluated the overall predictive accuracy in terms of the likelihood of predicted distributions to examine the relevance of the predictive models for the component parameters and the dependence on prediction times.

In the first experiment (i.e. DDMM parameter prediction), we estimated the DDMM parameters using the music data created before and in a referential year *t*_0_ by the method presented in §3.2. We then predicted the model parameters for later years using the method presented in §3.3. This prediction method will be referred to as the state-space evolutionary model (SSEM). For comparison, we refer to a method that simply uses as predictions the model parameters estimated for the referential year as the ‘static model’. We also compared the predictions made by the Gaussian process (GP) model [44], which is a general-purpose regression method, and in particular, a generalization of the linear regression model. We used a multi-kernel GP composed of a linear kernel, Gaussian kernel, Matérn kernel with degree *ν* = 5/2, and a bias term. When applying the SSEM and GP to predict the values of the mean distributions, we used these methods only for components with an average probability in the past data above a threshold of 0.01 and used the static model for the other rare components. This is because the probabilities of rare components are susceptible to large statistical noise and cannot be reliably estimated. The weights and kernel parameters of the GP model were optimized to predict each DDMM parameter (the GPy library [45] was used in the analysis). To evaluate the predictive ability, we computed the errors for each set of DDMM parameters. We used the SKL divergence to measure the prediction errors for the mixture probabilities and mean distributions, and used the log-squared error for the concentration parameters.

**Figure 5.**
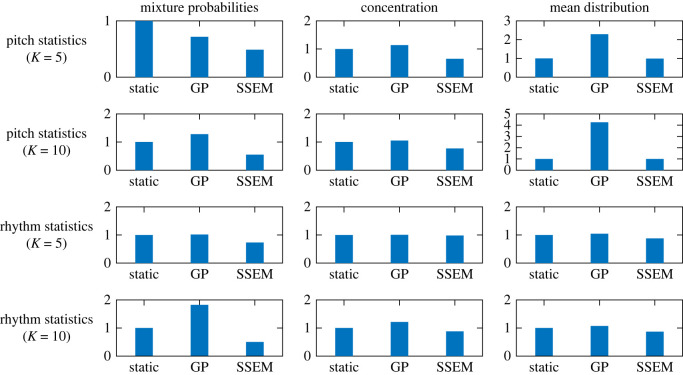
Errors of DDMM parameter prediction by the static model, Gaussian process (GP), and the state-space evolutionary model (SSEM). All errors are normalized by the values for the static model.

The results are summarized in figure 5, where the aforementioned three methods are compared for both pitch and rhythm statistics for the range *t*_0_ ∈ [1969, 2009]. For the mixture probabilities, the SSEM had significantly smaller errors than the static model in all cases. The GP model had larger errors than the static model, except for the pitch statistics and the number of clusters *K* = 5. For the concentrations, the SSEM had smaller errors than the static model, except for the case of rhythm statistics and *K* = 5, where the errors for these models were similar. The GP model had a similar or larger error than the static model for all cases. For the mean distribution parameters, the SSEM and the static model had similar errors in the case of pitch statistics, while the former model had slightly smaller errors in the case of rhythm statistics. The GP model had the largest errors in all cases.

Overall, the GP model often had larger prediction errors than the static model, indicating that the prediction of the DDMM parameters is a non-trivial problem. In most cases, the SSEM can predict the parameters more accurately than the static model. In particular, the reduction in error in the mixture probability predictions was considerably large, suggesting the effectiveness of describing the dynamics by a fitness-based evolutionary process with smoothly varying fitness values for the clusters. However, the error reductions in the mean distribution parameters were relatively small, implying a possibility that the parameters follow more complicated dynamics.

**Figure 6.**
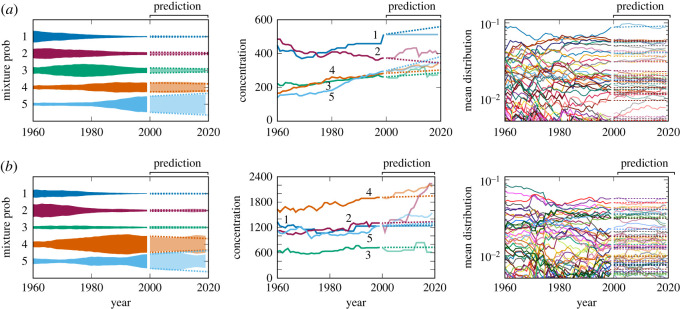
Examples of predicted and actual evolutions of the DDMM parameters of the (*a*) pitch and (*b*) rhythm statistics. After the reference year *t*_0_ = 1999, actual evolution curves are displayed in pale colours and predicted ones are displayed by dotted curves. The rightmost panel shows the mean distributions of the fifth cluster.

Examples of predicted and actual evolutions of the DDMM parameters of the pitch and rhythm statistics, when the reference year was set to *t*_0_ = 1999 and the number of clusters was *K* = 5, are shown in figure 6. For the mixture probabilities and concentrations, the SSEM linearly extrapolates recent trends and in most cases succeeds in predicting the directions of evolution. For the mean distributions, the predictions are conservative and only slightly differ from constant values. Significant errors are observed especially for musical elements with small probabilities.

**Figure 7.**
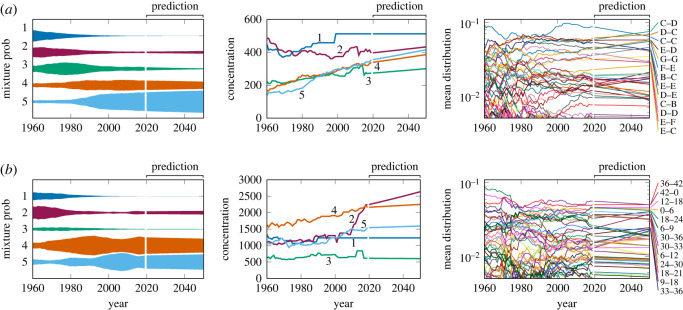
The predicted evolution of the DDMM parameters of the (*a*) pitch and (*b*) rhythm statistics. The rightmost panel shows the mean distributions of the fifth cluster, and the labels on the right-hand side indicate the corresponding bigrams.

The future values of the DDMM parameters predicted by the SSEM are shown in figure 7 for the case with *K* = 5 clusters. To make it easier to examine trends, the fitness reduction factor was set to unity for the mean distribution parameters to obtain these results. For both pitch and rhythm statistics, it is predicted that the fifth cluster will continue to rise and become dominant. For rhythm statistics, the second cluster is also predicted to rise in the near future. The concentrations for all rising clusters are predicted to increase, indicating a decrease in the variabilities within individual clusters.

Focusing on the fifth cluster of the pitch statistics, some changes in the probabilities of the most frequent bigrams are predicted. In particular, the probabilities of stepwise motions, such as (C,D), (F,E) and (B,C), are predicted to increase, whereas those of same-tone motions, such as (C,C), (E,E) and (D,D), are predicted to decrease. For the fifth cluster of the rhythm statistics, the probabilities of currently the most frequent bigrams are predicted to eventually decrease, indicating a general uniformization of bigram probabilities. Notable exceptions are bigrams with a 16th-note length, such as (6,9), (30,33) and (33,36), for which relatively rapid increases in the probabilities are predicted. The observed trends in the to-be-dominant clusters of the pitch and rhythm statistics are consistent in reducing monotonic motions (same-tone or eighth note rhythm). To illustrate the effect of these parameter changes, some melodies generated by the mean distributions observed in 2019 and those predicted in 2040 are given in electronic supplementary material. Perceptually, however, it is difficult to clearly distinguish the styles of these melodies.

In the second experiment (i.e. prediction of the creation style distributions), we measured the likelihood of the probability vectors in a range of 20 years after a referential year *t*_0_, using the parameters predicted by the SSEM. For predicting the creation style distribution at year *t*_*p*_ after *t*_0_, we used the parameters predicted for year *t*_*p*_ + Δ_*p*_, where Δ_*p*_ is a ‘look-ahead’ parameter that compensates for the effect of the weighted average smoothing. The value of Δ_*p*_ was optimized to maximize the likelihood, resulting in values Δ_*p*_ = 8 and 15 for the pitch and rhythm statistics, respectively. For the same purpose, we also used increased values for concentrations because the creation style distribution of an individual year was expected to have a larger concentration (smaller variance) than the smoothed one. For the analysis, an increasing factor of 5 was used for all data. For comparison, we measured the likelihood of the same data using the static model explained above. We evaluated cases *K* = 5 and 10 for the number of clusters, and used the static model with a single cluster (*K* = 1) as the baseline.

**Figure 8.**
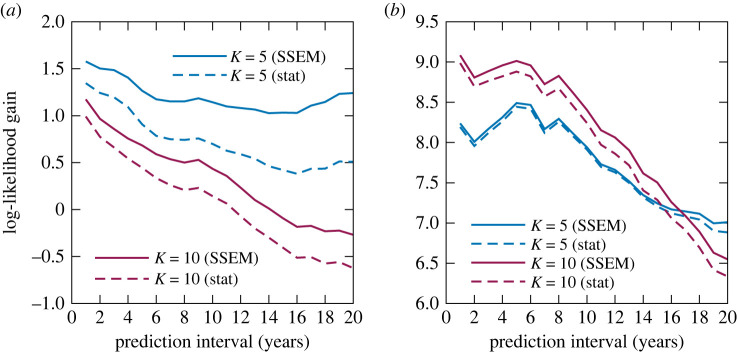
The log-likelihood gains obtained by the SSEM and static model for the (*a*) pitch and (*b*) rhythm statistics.

Figure 8 shows the dependence of the log-likelihood gains (compared with the *K* = 1 static model) on the predictive intervals (which is defined as the year of prediction—the referential year), averaged over all referential years in the range *t*_0_ ∈ [1969, 2009]. First, in all cases, the SSEM had a larger likelihood than the static model, indicating that the DDMM parameter prediction made by the SSEM can assist in predicting the distribution of the creation styles up to a prediction interval of at least 20 years. Second, for both the pitch and rhythm statistics, the likelihood gains of the models with *K* = 10 tended to decrease for longer prediction intervals and fell beneath the gains of the model with *K* = 1 (pitch statistics) or *K* = 5 (rhythm statistics) for predictive intervals of nearly 20 years. This result indicates the difficulty of predicting the distribution increases for large numbers of clusters. In addition to the possible effects of overfitting and data sparseness, the increase in the number of parameters that are difficult to predict might have led to the deterioration of the likelihood gains.

**Figure 9.**
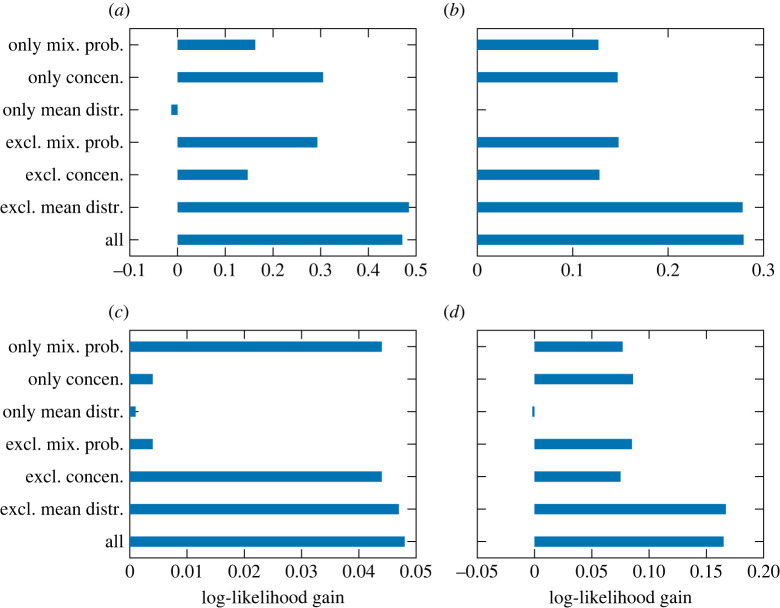
The contributions of individual elements of the DDMM parameters to the log-likelihood gains. (*a*) Pitch statistics (*K* = 5), (*b*) pitch statistics (*K* = 10), (*c*) rhythm statistics (*K* = 5) and (*d*) rhythm statistics (*K* = 10).

Finally, to examine the contributions of individual elements of the DDMM parameters to the likelihood gains, we compared the gains obtained by using the predictions by the SSEM for partial sets of the parameters and using the static model’s predictions elsewhere. The results in figure 9 show that the SSEM’s predictions of the mixture probabilities and concentrations contribute to the likelihood gains to some extent, and their contributions are often comparative. This indicates that the predictions of both inter- and intra-cluster dynamics have positive effects on likelihood gains. However, the SSEM’s predictions of the mean distribution parameters have small and sometimes even negative effects. Again, this is mainly due to the difficulty of predicting these parameters.

## 4. Case of US popular music

In this section, we analyse the US popular music data (US-pop dataset), developed by Mauch *et al.* [12], using the methods described in §§2 and 3. The dataset comprises 17 094 songs that appeared in the Billboard Hot 100 charts between 1960 and 2009. The audio clips of the songs were analysed to extract timbral and harmonic features. We analysed the lexicalized representations of these features obtained in [12], where each song was represented by sequences composed of 35 ‘T-lexicons’ and 193 ‘H-lexicons’, respectively, and used their unigram probabilities as timbre and harmony statistics.

**Figure 10.**
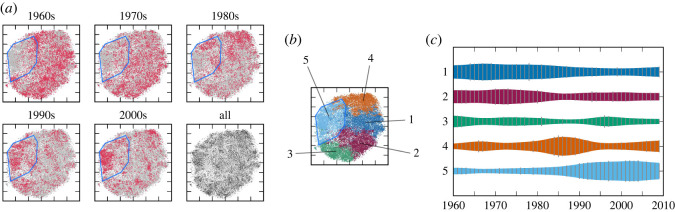
The evolution of the distribution and cluster structure of the timbre statistics in the US-pop data. (*a*) The two-dimensional visualization of the data distribution (blue polygons outline regions corresponding to cluster 5). (*b*) Result of the cluster analysis. (*c*) The evolution of the relative frequencies of the clusters.

**Figure 11.**
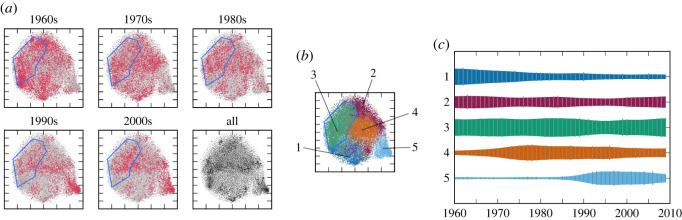
The evolution of the distribution and cluster structure of the harmony statistics in the US-pop data. (*a*) The two-dimensional visualization of the data distribution (blue polygons outline regions corresponding to cluster 3). (*b*) Result of the cluster analysis. (*c*) The evolution of the relative frequencies of the clusters.

The two-dimensional visualization and clustering with *K* = 5 classes in figures 10 and 11 show that the creation style distributions changed considerably during the analysis period, and that the cluster structure was not very clear in these data. While the overall changes can be represented as the evolution of relative frequencies of the clusters, we can also observe intra-cluster dynamics in both data. For example, cluster 5 of the timbre statistics contracted between the 1990s and the 2000s, while cluster 3 of the harmony statistics shifted its density distribution between the 1960s and the 1990s.

**Figure 12.**
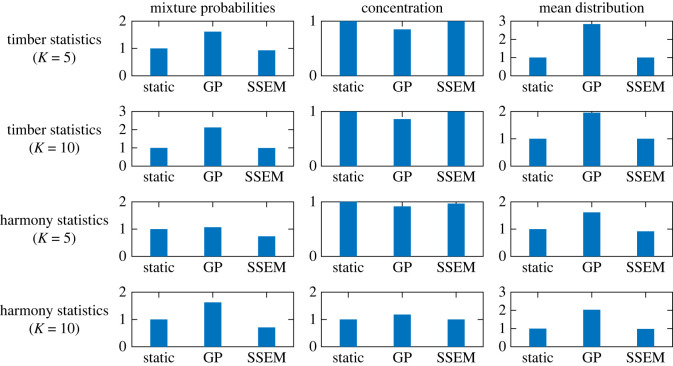
Errors of the DDMM parameter prediction by the static model, Gaussian process (GP), and the state-space evolutionary model (SSEM) for the US-pop dataset. All errors are normalized by the values for the static model.

Next, we studied the prediction of future creation style distributions using the method described in §3. The experimental set-ups were the same as in §3.4, and the referential year was set in the range *t*_0_ ∈ [1979, 1999]. The results of the DDMM parameter prediction using the SSEM in figure 12 show tendencies similar to those observed in figure 5 for the J-pop dataset. Overall, the SSEM improved the prediction accuracy compared with the static model. Additionally, the predictions by the GP were often less accurate than those of the static model, indicating the non-trivial nature of the prediction problem.

**Figure 13.**
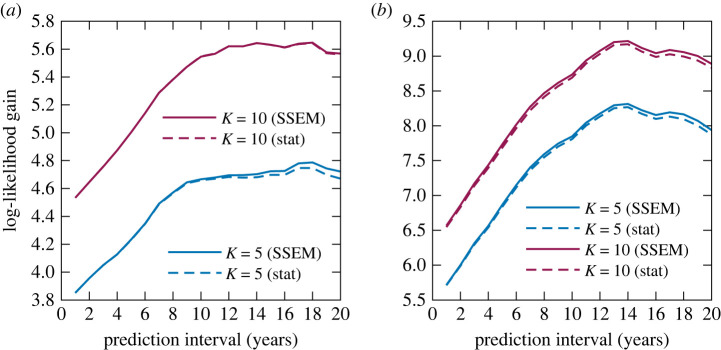
The log-likelihood gains obtained by the SSEM and static model for the (*a*) timbre and (*b*) harmony statistics in the US-pop dataset.

**Figure 14.**
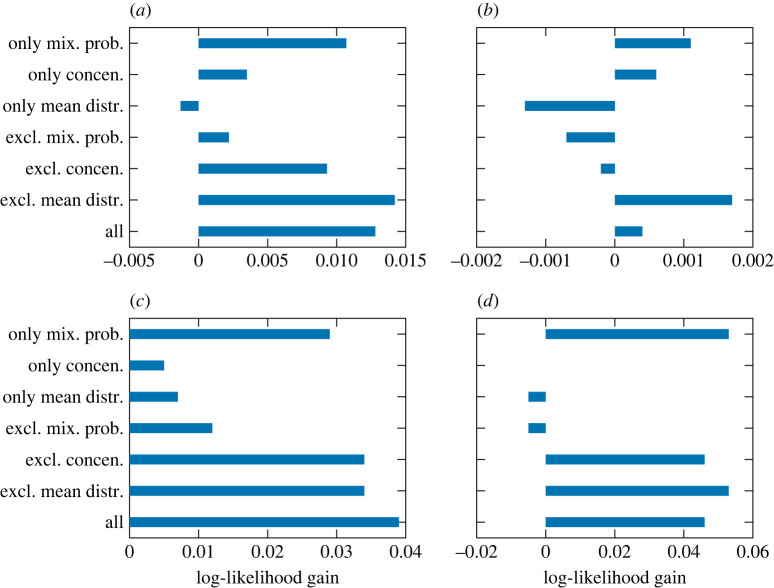
The contributions of individual elements of the DDMM parameters to the log-likelihood gains for the US-pop dataset. (*a*) Timbre statistics (*K* = 5), (*b*) timbre statistics (*K* = 10), (*c*) harmony statistics (*K* = 5) and (*d*) harmony statistics (*K* = 10).

The results of the likelihood evaluation (figure 13) and the contributions of individual elements of the DDMM parameters to the likelihood gains (figure 14) indicate positive effects of the DDMM parameter predictions by the SSEM and the relevance of both inter- and intra-cluster dynamics, which are qualitatively the same conclusions as in the case of the J-pop dataset. For the US-pop dataset, however, the log-likelihood gains obtained by the SSEM were relatively small, and using *K* = 10 clusters always led to a higher prediction accuracy than using *K* = 5 clusters. The latter result can be ascribed to the large number of samples and more complex structure of the creation style distributions in this dataset.

## 5. Discussion and conclusion

In this study, we have investigated the evolution of music creation style distributions using statistical modelling methods. By analysing clusters found in several musical statistics extracted from the J-pop and US-pop datasets, we identified intra-cluster dynamics, such as the contractions of clusters’ sizes and shift of clusters’ centres, as well as prominent inter-cluster dynamics in the relative frequencies of clusters. These different modes of cluster dynamics were incorporated into the DDMM in a unified manner. We then used the DDMM to evaluate its predictive ability and found that both inter- and intra-cluster dynamics are relevant for predicting future creation style distributions. In particular, the prediction of cluster variances often contributed to the likelihood gain more than the prediction of cluster frequencies in the analysis of the J-pop dataset. These results highlight the importance of analysing cluster variances for characterizing and predicting the evolution of creation style distributions, in addition to analysing the frequencies and means of clusters, which have been the focus of most previous studies on quantitative cultural evolution analysis [12,15,46,47].

It should be remarked that we used datasets each covering a small proportion of popular music songs and the distributions of creation styles we analysed represent those of hit songs. Previous studies have shown that songs in the top chart and those out of the top chart have significantly different distributions and trends of music styles [15], and that changes in the dominant styles of popular music can arise from the styles of previously minor songs with very different characteristics [48]. Those results suggest that the evolution of music styles of hit songs is not a closed system and analysis of data spanning a wider range of popularity may also be useful for predicting the style evolution of hit songs.

Our results open new possibilities regarding the quantitative and theoretical understanding of cultural evolution. First, as the results indicate that different clusters in the space of creation styles can have different modes of intra-cluster dynamics, future research should further investigate the causes of differences in the characteristics of these dynamics. Since a cluster in cultural data often corresponds to a stylistic genre of a culture, which often involves a specific community in a society, the type of the community’s public preferences, such as preferences towards typicality or novelty [16], might be relevant.

Second, from figures 1, 2, 10 and 11, clusters of creation styles have a hierarchical structure and significantly overlap with each other. In this situation, it is difficult to define the number of clusters, and therefore, the distinction between inter- and intra-cluster dynamics is essentially relative. While using a fixed number of clusters for analysis, as in this study, often facilitates the interpretation of dynamics, another possibility is to apply the non-parametric Bayesian framework [49,50] to consider all possible numbers of clusters. As we have confirmed the concurrent and transient cluster structures of creation styles, it would be interesting to compare the present method (dynamic analysis after clustering) with the methods for jointly conducting clustering and dynamic analysis [51,52]. These topics will be investigated in future research.

Third, using the SSEM, predicting mean distributions is often more difficult than predicting the mixture probabilities and concentrations. A possible reason for this is the high dimensionality of the data space of creation styles. Additionally, from the analysis of the J-pop dataset (figure 7), the evolutions of the component statistics often have musical relations and are not totally independent. This suggests that the effective dimension of evolution is much lower than the feature dimension, similar to some observations in the biological evolution of phenotypes [53,54]. Thus, an important problem that will be addressed in future work is to estimate the effective space of evolution from the data and construct an evolutionary model in the reduced space.

Fourth, the constructed evolutionary model represents a simplified description of the underlying process of knowledge transmission and selection, and can be extended to study more detailed dynamics of music style evolution. Among many possible ways of model extension, one is to explicitly include ‘creators’, each of which can be represented by a collection of statistics corresponding to multiple songs it produces. This extension enables to distinguish artefact-level selection and creator-level selection, and to study the effect of possibly intricate relationship between creators and clusters of song-level statistics. An extension to include a process of learning from multiple songs possibly from more than one previous generation is important to make the knowledge transmission process more realistic, and it enables to study how the distribution of learning samples, both in time and in the space of music styles, affects lineage-level and population-level evolutions. For empirical validation, it is also important to examine how such refined models can improve the ability of predicting future creation style distributions, and the present model can be used as a baseline model for quantitative comparisons.

Finally, although simple statistics such as pitch and rhythm bigrams are analysed in this study, more complex statistics, such as higher-order ngram probabilities and joint probabilities of pitch and rhythm, can be used to better approximate the generative process of music data. The present method, which uses the Dirichlet distribution to model clusters of probability vectors, can be applied to cultural artefacts whose generative process can be characterized by a discrete probability distribution. An extension to deal with probabilistic models with latent variables and neural-network-based generative models is also important since they are used in high-quality automatic music generation systems [20]. Since probabilistic generative models are ubiquitously used for digitized cultural data, the proposed methodology can potentially be applied to other domains of creative culture, such as lyrics [55], visual art [56], scientific papers [57] and culinary art [58], where dynamic cluster structure and content-based selection play vital roles. An advantage of analysing the evolution of the statistics of a probabilistic generative model is that it enables not only the analysis and prediction of content features but also the generation of synthetic cultural artefacts in the styles of the past, present and future, in line with the analysis-by-synthesis framework [59]. This approach can, therefore, be applicable for realizing a ‘creative’ system for music generation, rather than only a system imitating the style of existing artefacts, which is a major problem in automatic music generation systems based on machine learning.

## Data Availability

The music statistics data and source code used for the analysis, the data obtained by the analysis and melody audio samples are available in electronic supplementary material [60].
